# Strengths-based resilience: the biopsychosocial factors that differentiate 12-year mental well-being trajectories following adverse childhood experiences

**DOI:** 10.1017/S0033291726104784

**Published:** 2026-06-09

**Authors:** Elizabeth Connon, Haeme R.P. Park, Robin M. Turner, Leanne M. Williams, Justine Megan Gatt

**Affiliations:** 1 School of Psychology, University of New South Wales, Sydney, NSW, Australia; 2 Neuroscience Research Australia, Randwick, NSW, Australia; 3 Department of Public Health, University of Otago, Dunedin, Central Dunedin, New Zealand; 4 Stanford University School of Medicine, Stanford, California, USA

**Keywords:** ACEs, biopsychosocial factors, childhood trauma, COMPAS-W, longitudinal trajectories, mental well-being, resilience

## Abstract

**Background:**

Adverse childhood experiences (ACEs) are associated with increased long-term mental and physical health risk, yet inter-individual variation indicates that resilience is possible. Distinct trajectories of sustained mental well-being have been observed among individuals exposed to ACEs, predicting favorable long-term outcomes. However, the biopsychosocial markers underpinning sustained trajectories of resilience remain poorly understood. This study aimed to identify factors that predict higher well-being (‘resilient’) trajectories over time in individuals with and without ACE exposure.

**Methods:**

Previously identified 12-year well-being trajectory classes (higher vs. lower well-being) were examined in participants with no ACE history (*n* = 779) and those exposed to ACEs (*n* = 889). Logistic regression models were used to identify factors differentiating higher versus lower well-being trajectories in the non-ACE sample, and resilience versus risk trajectories in the ACE sample. Predictors included childhood adversity characteristics, genetic predisposition, demographic and environmental factors, social and occupational factors, health and lifestyle factors, and psychological functioning.

**Results:**

Across both samples, higher well-being was predicted by higher education, better subjective physical health, greater social engagement, stronger work performance, and adaptive personality traits (lower neuroticism and higher extraversion). In the non-ACE sample, polygenic well-being scores and family history of mental illness further differentiated trajectories. In contrast, resilience following ACEs was additionally characterized by modifiable factors, including parenting style, relationship status, BMI, and conscientiousness.

**Conclusions:**

Resilience in the context of childhood adversity is defined by identifiable, largely modifiable social, health, and personality factors, highlighting potential targets for interventions to enhance long-term well-being.

## Introduction

Research consistently demonstrates that adverse childhood experiences (ACEs) are a prevalent, transdiagnostic risk factor for poor mental and physical health (Hughes et al., 2017). ACEs occur during critical periods of social learning and physiological development, and thus exert enduring negative effects across the lifespan (Bossert, Jayawickreme, Blackie, & Cole, 2024; Ho & King, 2021). However, individuals show marked variability in psychological and behavioral responses to adversity, indicating that resilience is possible (Bonanno & Westphal, 2024). Consistent with this, prior work has identified distinct mental well-being trajectories over a 12-year period, including a resilient subgroup who maintained higher well-being despite ACE exposure (Connon et al., 2026). Compared to those with lower well-being, these resilient individuals exhibited health and lifestyle benefits lasting across adulthood. However, understanding of the biopsychosocial factors that differentiate and support such resilience is lacking, yet is critically needed to actively promote positive outcomes among vulnerable ACE-exposed individuals.

At present, research examining long-term resilience in the context of ACEs remains fragmented and marked by inconsistent empirical findings. Establishing whether relatively stable biological factors contribute to resilience is a necessary foundational step to clarifying which influences must be accounted for before targeting more malleable determinants of well-being in effective interventions. Evidence regarding age remains mixed. Some studies suggest that younger individuals are more vulnerable to the negative consequences of ACE exposure (Mugoya et al., 2024), whereas other work indicates ACEs exert persistent detrimental effects across the lifespan, increasing risk for mental distress even in older adulthood (Liu, Cao, & Wei, 2025). Sex differences add further complexity, as the negative effects of ACEs have been found to manifest differently in males and females (Curran et al., 2018; Mugoya et al., 2024). Family history of mental illness also elevates transdiagnostic risk (Uher et al., 2023), with evidence that ACEs may exacerbate this vulnerability (Streit et al., 2023). Finally, biological genetic factors have been linked to mental health and resilience, leading to the creation of polygenic scores that summarize an individual’s genetic propensity for a given trait, including those representing higher well-being (de Vries et al., 2021; Jamshidi et al., 2020). However, the association between such scores and complex, multidimensional constructs like well-being is typically small and indirect (Chabris et al., 2015), and their predictive utility is likely to depend on environmental context. Consistent with gene–environment interaction frameworks (Halldorsdottir & Binder, 2017), it is plausible that genetic influences on well-being may be differentially expressed across levels of adversity. In lower-adversity contexts, genetic predispositions may be more readily observable, whereas in the presence of significant early adversity, environmental factors may constrain or attenuate their expression. This raises the possibility that pathways to resilience differ between ACE-exposed and nonexposed individuals.

Beyond biological influences, social and environmental factors play a critical role in shaping resilience following ACEs. While a broad range of ACEs have been associated with elevated psychopathology (Sasaki et al., 2024), evidence suggests that specific exposures – particularly abuse and interpersonal conflict – may confer disproportionately harmful effects and, therefore warrant heightened attention (Lee et al., 2024). These adverse effects appear to be further compounded by low levels of supportive parenting (Anderson et al., 2022). Education has also been implicated in resilience, although findings remain mixed: while some studies suggest that higher educational attainment predicts more resilient outcomes (Buchanan et al., 2023), others report a neutralizing effect whereby increased expectations may offset potential mental health benefits (Kristoffersen, 2018). In contrast, evidence is more consistent for the protective role of intimate relationships, with those in cohabiting partnerships associated with reduced psychological distress following ACEs (Mugoya et al., 2024). Perceived social support more broadly has similarly been linked to attenuating the relationship between ACEs and mental illness (Templeton et al., 2025). Extending to the wider social environment, ACE exposure has been associated with reduced work engagement (Herbert et al., 2023), though how this impacts mental health remains unclear.

Markers of physical health, including body mass index (BMI) (Doom et al., 2023), sleep quality (Sheffler et al., 2024), and adverse physiological or somatic symptoms (Eilers, aan het Rot, & Jeronimus, 2023) have also been implicated in the relationship between ACEs and poor mental health outcomes. Lifestyle behaviors appear to play a key intersecting role in these pathways, with evidence linking ACE exposure to poorer diet, reduced physical activity, and increased substance use (Yang et al., 2024). For instance, lower levels of physical activity have been shown to exacerbate the association between ACEs and mental distress (Wang et al., 2025), while ACE exposure has been associated with poorer eating habits (Aquilina et al., 2021), which in turn predict worse mental health outcomes (Collins et al., 2022). Similarly, intersecting associations between ACEs, substance use, and mental ill-health have been consistently reported (Swedo et al., 2025).

Lastly, beyond health behaviors, psychological factors also appear central. Longitudinal studies indicate that personality traits play both mediating and moderating roles in the link between ACEs and mental ill-health, with neuroticism conferring risk and conscientiousness and extraversion offering protection (Li et al., 2023; Yuan et al., 2024). ACEs have also been linked to poorer emotional regulation, which in turn is associated with increased risk of mental illness and impaired resilient responding (Milojevich, Norwalk, & Sheridan, 2019; Weissman et al., 2019).

Despite these advances, the existing literature is subject to several important limitations. First, although resilience implies *positive* adaptation, most prior studies operationalize resilience indirectly as the absence of mental illness or distress, rather than as the presence of positive mental well-being (Hiebel et al., 2021). This approach is increasingly challenged by evidence supporting a dual-continua model of mental health, in which well-being and mental illness represent related but distinct processes (Lam, Park, & Gatt, 2024; Mason Stephens et al., 2023). This deficit-focused approach has constrained investigation into the factors that specifically predict higher well-being and actively promote positively defined resilience beyond symptom minimization (Meng et al., 2018; Yule et al., 2019). Second, much of the existing research has examined putative resilience factors in isolation and in disparate samples, limiting the ability to account for interacting influences within and across biopsychosocial domains (Latham, Newbury, & Fisher, 2023). The lack of concurrent, multivariable assessment precludes direct comparison of predictors, leaving their relative and independent contributions unclear. Third, ACE research has largely focused on younger cohorts assessed at single time points, with relatively few studies employing longitudinal designs that track resilience across adulthood. Long-term assessment is essential to identify the predictors of resilience that are sustained over time, and therefore represent meaningful targets for intervention (Infurna, 2021).

To address these limitations, we leveraged 12-year trajectories of higher and lower mental well-being previously identified across adulthood in individuals with and without exposure to childhood adversity (Connon et al., 2026). Using these resilience-risk (ACE sample) and high-low well-being (non-ACE sample) trajectories, we aimed to identify the biopsychosocial characteristics associated with well-being and resilience. We examined baseline adversity characteristics, biological (polygenic scores), demographic and environmental (age, sex, parenting, and education), social and occupational (relationship status and work performance), physical health and lifestyle (adverse symptoms, BMI, sleep, diet, and exercise), and psychological factors (emotion regulation and personality traits) to determine which features differentiated sustained trajectories across adulthood.

## Method

The present study draws on data from the TWIN-10 project, a longitudinal cohort study that followed participants over a 12-year period (Gatt et al., 2012; Park, Williams, Turner, & Gatt, 2022). Additional details are provided in the Supplementary Material and in Connon et al. (2026).

### Participants

Same-sex adult twins were recruited through Twins Research Australia. To minimize population stratification in genetic analyses, the sample was restricted to individuals of European ancestry. To characterize well-being within a nonclinical population, exclusion criteria included current or lifetime psychiatric illness, stroke or neurological disorder, genetic disorder, brain injury, chronic or serious medical conditions, blood-borne illnesses, or substance abuse. Proportional recruitment across ages 18–62 years ensured representation across adulthood. All participants with ACE data were included (*N* = 1668; mean [SD] age, 39.7 [12.7] years; 504 females [57%]). Written informed consent was obtained. Ethical approval was granted by the Human Research Ethics Committees of the University of Sydney (03–2009/11430) and the University of New South Wales (HC14256, HC180403).

### Well-being trajectories in response to ACE exposure

Well-being was assessed at four timepoints across 12 years using the COMPAS-W, a comprehensive scale developed through factor analysis of a broad range of hedonic and eudaimonic well-being measures. Capturing well-being across six sub-domains (Composure, Own-worth, Mastery, Positivity, Achievement, and Satisfaction), the COMPAS-W demonstrates strong internal consistency (*α* = 0.84) and test–retest reliability (0.82; Gatt et al., 2014).

ACEs were assessed retrospectively at baseline (T1) using the Early Life Stress Questionnaire (ELS-Q; Cohen et al., 2006), with demonstrated internal and criterion validity (Sokolowski & Dragan, 2017). The ‘Premature birth’ item was excluded due to high prevalence in twin births and nonpsychological relevance. Participants were classified as ACE-exposed (≥1 endorsed adversity; *n* = 889) or nonexposed (*n* = 779).

**Figure 1.**
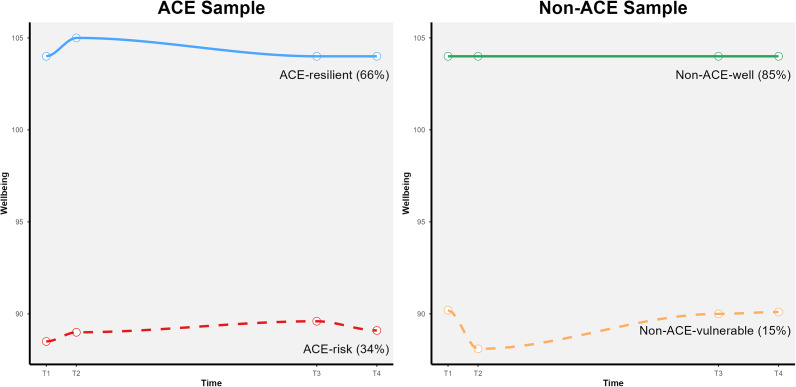
Well-being trajectories across 12 years among individuals with and without exposure to adverse childhood experiences (ACEs). *Note*: Trajectory membership was identified using longitudinal growth mixture modeling, as previously reported by Connon et al. (2026). Trajectories are shown here using newly generated smoothed estimates for visualization. Among participants exposed to ACEs, 65.8% (*n* = 585) were classed within an ACE-resilient trajectory, while 34.2% (*n* = 304) followed an ACE-risk trajectory. In the non-ACE sample, 84.7% of participants (*n* = 660) were classified within a non-ACE-well trajectory, and 15.3% (*n* = 119) within a non-ACE-vulnerable trajectory. ACE, adverse childhood experience. Figure 1. long description.

Longitudinal growth mixture modeling (LGMM) was applied to COMPAS-W scores to identify latent trajectory classes. As shown in Figure 1, two resilience-risk trajectories were identified within the ACE sample; a higher well-being ‘ACE-resilient’ trajectory (66%) and a lower well-being ‘ACE-risk’ trajectory (34%). Two higher–lower well-being trajectories were also identified within the non-ACE sample; the majority fell into the higher well-being ‘non-ACE well’ (85%), with a smaller portion classed as the lower well-being ‘non-ACE-vulnerable’ (15%). For further details, see Connon et al. (2026).

### Biopsychosocial predictors

#### ACE childhood adversity predictors

To identify distinct co-occurring patterns of childhood adversity and examine which forms of ACEs are most strongly related to resilience, a categorical principal component analysis (CATPCA) was conducted on ELS-Q items within the ACE sample. Derived ACE subtypes were entered as predictors of trajectory membership. See the Supplementary Methods for a detailed description of this analysis.

#### Genetic predictors

Polygenic well-being scores previously derived for the TWIN-10 sample were used (Jamshidi et al., 2020). These scores index genetic predisposition toward variation in mental well-being, operationalized using COMPAS-W, which captures hedonic and eudaimonic aspects of well-being.

#### Demographic and environmental predictors

Baseline measures of age, biological sex, family history of mental illness, and highest educational attainment were assessed as previously described (Gatt et al., 2012). Maladaptive parenting was assessed using the Measure of Parenting Style (MOPS; Cronbach’s *α* > .75; Parker et al., 1997).

#### Social and employment predictors

Relationship status (in a relationship vs not) and weekly social participation were assessed via self-report. Employment outcomes were measured using the World Health Organization Health and Work Performance Questionnaire (HPQ; Kessler et al., 2004), assessing absenteeism and self-rated work performance ( *α* = .74; Golz, Gerlach, Kilcher, & Peter, 2022).

#### Health and lifestyle predictors

Body mass index (BMI) was calculated from self-reported height and weight. Sleep duration, alcohol consumption, problematic substance use, smoking status, physical activity, and dietary intake were assessed via self-report. Subjective physical health was measured using the SPHERE (*α* > .85; Wijeratne, Hickie, & Davenport, 2006), and sleep quality using items from the Survey Screen for Apnea (*α* = .85–.93; Maislin et al., 1995).

#### Psychological predictors

Locus of control was assessed using the Internal Control Index (ICI; *α* = .84; Duttweiler, 1984). Personality traits were measured using the NEO Five-Factor Inventory (McCrae & Costa, 1987). Emotion regulation strategies were assessed using the Emotion Regulation Questionnaire (*α* > .73; Gross & John, 2003).

### Statistical analysis

Multivariate logistic regression models examined baseline predictors of higher versus lower well-being trajectory membership separately within the ACE and non-ACE samples. Odds ratios (ORs) represent the change in likelihood of membership in the higher well-being trajectory per unit increase in each predictor. Analyses were conducted in two stages: domain-specific models were first estimated, followed by combined cross-domain models including only predictors significant at *p* < .05 within domain analyses. This approach reduced overfitting and identified independent predictors of trajectory membership within each subsample. Within each domain-specific model and the final combined models, the Benjamini–Hochberg false discovery rate (FDR) procedure (Cai & Chan, 2017) was used to control for multiple comparisons, with statistical significance determined based on adjusted *p*-values (*p* < .05). All analyses were performed in R (version 4.4.2; R Core Team, 2025) and adjusted for age, biological sex, and zygosity, with family ID included as a random effect to account for twin relatedness. Models including polygenic scores were additionally adjusted for the first 10 genetic principal components.

## Results

Baseline demographic characteristics for the ACE- and non-ACE samples are presented in Supplementary Table 2. The groups did not differ in age, sex, relationship status, educational attainment, or family history of mental illness (all *p* ≥ .05). However, the ACE sample exhibited significantly higher maladaptive parenting scores (MOPS mother, *t*(1667) = −9.83, *p* < .001; father, *t*(1667) = −11.2, *p* < .001), while the non-ACE sample had higher baseline COMPAS-W well-being scores (*t*(1667) = 6.46, *p* < .001).

### ACE childhood adversity subtypes

The CATPCA of ELS-Q items yielded five components: ‘Family breakup’, comprising family separation and sustained conflict; ‘Interpersonal violation’, comprising physical abuse, emotional abuse, sexual abuse, and domestic violence; ‘Personal health trauma’; ‘Family illness, death, and disaster’; and ‘Peer conflict’, referring to sustained bullying or rejection by schoolmates. These components explained 55.53% of the variance (see Supplementary Table 1 for further details).

### Prediction of trajectory membership

Results from the domain-specific multivariate logistic regression models are presented in Table 1 for the ACE sample and Table 2 for the non-ACE sample.

**Table 1.** Within-domain associations between biopsychosocial factors and well-being trajectories (Resilient vs. Risk) in the ACE sample (*N* = 889) Table 1. long description.

Domain	Variable	Resilient (vs. Risk)		
		Odds ratio	95% CIs	*p*-value
Childhood adversity subtypes	Interpersonal violation	**0.60**	**0.43, 0.85**	**0.003**
	Peer conflict	**0.64**	**0.47, 0.86**	**0.004**
	Family separation	0.96	0.69, 1.33	0.80
	Personal health trauma	0.78	0.54, 1.13	0.19
	Family illness/death/disaster	0.87	0.81, 1.24	0.45
	Cumulative count	**0.88**	**0.80, 0.96**	**0.006**
Genetic predisposition	Polygenic score	1.00	0.97, 1.03	0.85
Demographic and environmental factors	Age (years)	1.12	0.96, 1.32	0.16
	Sex (*reference = male*)	1.29	0.92, 1.81	0.14
	Family history of mental illness	0.65	0.46, 1.01	0.11
	Educational attainment	**1.35**	**1.15, 1.57**	**<.001**
	MOPS – Mother	**0.96**	**0.93, 0.99**	**0.009**
	MOPS – Father	**0.97**	**0.95, 0.99**	**0.03**
Social and occupational factors	Relationship status *(reference = in a relationship)*	**2.23**	**1.56, 3.18**	**<.001**
	Socializing with friends	**1.47**	**1.18, 1.84**	**<.001**
	Socializing with family	0.96	0.83, 1.11	0.60
	Absenteeism (HPQ)	1.00	0.99, 1.00	0.21
	Work performance (HPQ)	**1.35**	**1.18, 1.53**	**<.001**
Health and lifestyle indicators	BMI category	**0.73**	**0.59, 0.89**	**0.003**
	Hours slept *(reference = > 5 hours)*			
	5–8 hours	0.75	0.42, 1.32	0.32
	8+ hours	0.89	0.42, 1.87	0.76
	Alcohol intake *(reference = never)*			
	Low (monthly)	1.13	0.73, 1.76	0.57
	Moderate (weekly)	1.57	0.95, 2.60	0.08
	Frequent (4 or more times per week)	1.21	0.70, 2.09	0.48
	Substance use problems	0.73	0.41, 1.31	0.30
	Smoking status *(reference = never)*			
	Former	0.90	0.58, 1.38	0.62
	Current	0.70	0.42, 1.17	0.17
	Physical activity	1.04	0.90, 1.20	0.60
	Fruit and vegetable intake	1.14	0.95, 1.36	0.16
	Fast food intake	1.00	0.78, 1.28	0.98
	Sleep problems	1.01	0.99, 1.04	0.32
	Adverse physical health symptoms (SPHERE)	**0.89**	**0.86, 0.91**	**<.001**
Psychological factors	Locus of control (ICI)	**1.06**	**1.03, 1.08**	**<.001**
	Neuroticism (NEO-FFI)	**0.89**	**0.87, 0.92**	**<.001**
	Extraversion (NEO-FFI)	**1.15**	**1.10, 1.20**	**<.001**
	Openness (NEO-FFI)	**1.04**	**1.01, 1.08**	**0.02**
	Agreeableness (NEO-FFI)	1.01	0.96, 1.04	0.91
	Conscientiousness (NEO-FFI)	**1.07**	**1.03, 1.11**	**0.001**
	Reappraisal (ERQ)	1.01	0.98, 1.05	0.51
	Expressive suppression (ERQ)	1.01	0.97, 1.05	0.61

*Note*: Analysis adjusted for age, sex, zygosity, and family relatedness. Bolding indicates a statistically significant difference between the well-being trajectories. MOPS, Measure of Parenting Style; HPQ, Health and Work Performance Questionnaire; BMI, Body Mass Index; SPHERE, Somatic and Psychological HEalth REport; ICI, Internal Control Index; NEO-FFI, NEO Five Factor Inventory; ERQ, Emotion Regulation Questionnaire.

**Table 2.** Within-domain associations between biopsychosocial factors and well-being trajectories (Well vs. Vulnerable) in the non-ACE sample (*N* = 779) Table 2. long description.

Domain	Variable	Well (vs. Vulnerable)		
		Odds ratio	95% CIs	*p*-value
Genetic predisposition	Polygenic score	**1.09**	**1.04, 1.15**	**<.001**
Demographic and environmental factors	Age	1.01	0.99, 1.03	0.16
	Sex (*reference = male*)	0.75	0.46, 1.23	0.25
	Family history of mental illness	**0.53**	**0.29, 0.95**	**0.03**
	Educational attainment	**1.36**	**1.11, 1.68**	**0.004**
	MOPS – Mother	0.95	0.88, 1.02	0.20
	MOPS – Father	0.98	0.90, 1.04	0.50
Social and occupational factors	Relationship status (*reference = in a relationship)*	1.44	0.86, 2.41	0.16
	Socializing with friends	**1.53**	**1.12, 2.08**	**0.007**
	Socializing with family	0.85	0.70, 1.02	0.81
	Absenteeism (HPQ)	1.00	0.99, 1.00	0.45
	Work performance (HPQ)	**1.31**	**1.10, 1.56**	**0.003**
Health and lifestyle indicators	BMI (category)	1.26	0.91, 1.72	0.15
	Hours slept *(reference = > 5 hours)*			
	5–8 hours	1.10	0.41, 2.96	0.85
	8+ hours	1.06	0.24, 3.24	0.92
	Alcohol intake *(reference = never)*			
	Low (monthly)	1.61	0.92, 2.82	0.10
	Moderate (weekly)	**3.00**	**1.50, 5.99**	**0.002**
	Frequent (4 or more times per week)	1.67	0.82, 3.40	0.16
	Substance use problems	1.55	0.53, 4.57	0.42
	Smoking status *(reference = never)*			
	Former	1.06	0.54, 2.05	0.87
	Current	2.04	0.77, 6.20	0.21
	Physical activity	1.13	0.92, 1.39	0.26
	Fruit and vegetable intake	**1.41**	**1.11, 1.80**	**0.005**
	Fast food intake	0.83	0.57, 1.20	0.32
	Adverse physical health symptoms (SPHERE)	**0.90**	**0.86, 0.94**	**<.001**
	Sleep problems	0.99	0.95, 1.03	0.71
Psychological factors	Locus of control (ICI)	**1.06**	**1.03, 1.09**	**<.001**
	Neuroticism (NEO-FFI)	**0.91**	**0.88, 0.95**	**<.001**
	Extraversion (NEO-FFI)	**1.12**	**1.08, 1.18**	**<.001**
	Openness (NEO-FFI)	1.01	0.97, 1.05	0.57
	Agreeableness (NEO-FFI)	1.02	0.97, 1.08	0.42
	Conscientiousness (NEO-FFI)	1.03	0.98, 1.08	0.33
	Reappraisal (ERQ)	1.01	0.96, 1.06	0.66
	Expressive suppression (ERQ)	0.97	0.92, 1.02	0.23

*Note:* Analysis adjusted for age, sex, zygosity, and family relatedness. Bolding indicates a statistically significant difference between the well-being trajectories. MOPS, Measure of Parenting Style; HPQ, Health and Work Performance Questionnaire; BMI, Body Mass Index; SPHERE, Somatic and Psychological HEalth REport; ICI, Internal Control Index; NEO-FFI, NEO Five Factor Inventory; ERQ, Emotion Regulation Questionnaire.

**Figure 2.**
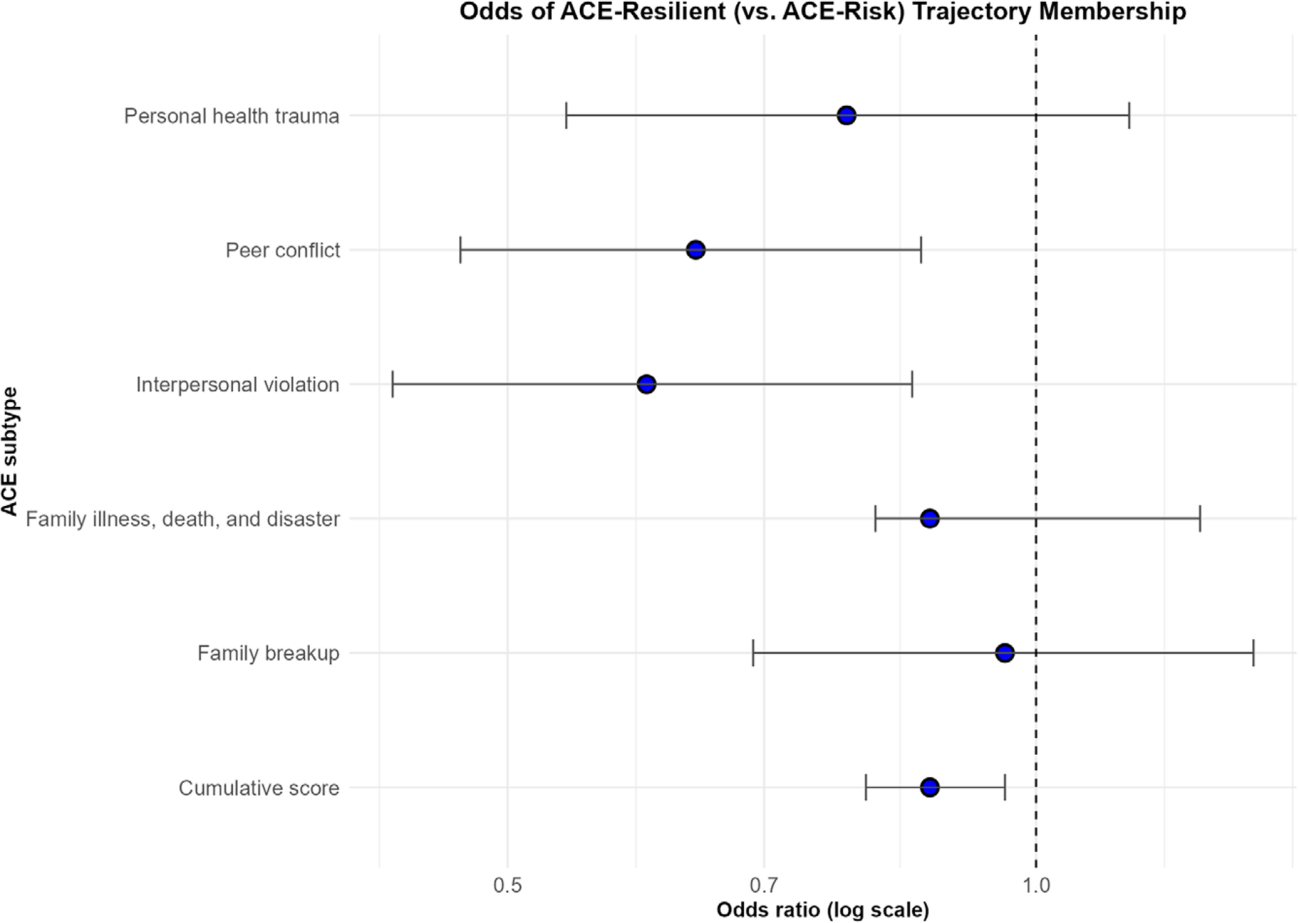
Associations between childhood adversity subtypes and well-being trajectories in the ACE sample. *Note*: Odds ratios (ORs) represent the likelihood of membership in the higher well-being trajectory (ACE-resilient vs. ACE-risk) associated with each form of adversity in the ACE sample. Plotted on a logarithmic scale, an OR of 1 indicates no association, shown by the dotted reference line at the 1 mark of the *x*-axis. ORs <1 indicate reduced odds, while ORs >1 indicate increased odds of membership in the higher well-being ‘ACE-resilient’ trajectory. Error bars represent 95% confidence intervals. Analyses were adjusted for age, sex, zygosity, and family relatedness. ACE, adverse childhood experience. Figure 2. long description.

Using childhood adversity subtypes derived from the CATPCA of ELS-Q items within the ACE sample, relational forms of adversity were negatively associated with resilience (see Figure 2). Exposure to *interpersonal violation* was associated with a 40% reduction in the odds of membership in the ‘ACE-resilient’ trajectory (OR = 0.60; 95% CI: [0.43, 0.85]), while *peer conflict* was associated with a 36% reduction (OR = 0.64; 95% CI: [0.47, 0.86]). Cumulative adversity also exerted a detrimental effect, with each additional ELS-Q event associated with a 12% decrease in the likelihood of ‘ACE-resilient’ trajectory membership (OR = 0.88; 95% CI: [0.80, 0.96]).

**Figure 3.**
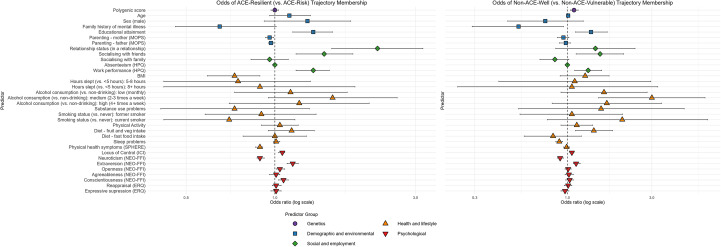
Associations between biopsychosocial predictors and well-being trajectory membership. *Note*: Odds ratios (ORs) represent the likelihood of membership in the higher well-being trajectory (‘ACE-resilient’ on the left panel and ‘non-ACE-well’ on the right panel) associated with each predictor, plotted on a logarithmic scale. An OR of 1 indicates no association, shown by the dotted reference line. ORs <1 indicate reduced odds, and ORs >1 indicate increased odds of membership in the higher well-being trajectory. Error bars represent 95% confidence intervals. All analyses were adjusted for age, sex, zygosity, and family relatedness. ACE, adverse childhood experience; MOPS, Measure of Parenting Style; HPQ, Health and Work Performance Questionnaire; BMI, Body Mass Index; SPHERE, Somatic and Psychological HEalth REport; ICI, Internal Control Index; NEO-FFI, NEO Five Factor Inventory; ERQ, Emotion Regulation Questionnaire. Figure 3. long description.

Genetic predisposition to well-being, indexed by polygenic scores, was not associated with trajectory membership in the ACE sample (see in Figure 3). In contrast, higher polygenic well-being scores were associated with increased odds of belonging to the ‘non-ACE-well’, compared to ‘non-ACE-vulnerable’, trajectory in the non-ACE sample (OR = 1.09; 95% CI: [1.04, 1.15]).

With respect to demographic and environmental factors, age and sex were not significant predictors of trajectory membership in either sample. However, in the non-ACE sample alone, a family history of mental illness was associated with a nearly 50% reduction in the odds of membership in the ‘non-ACE-well’ trajectory (OR = 0.53; 95% CI: [0.29, 0.95]). Early life environmental factors were influential in the ACE sample only: higher levels of maladaptive parenting were associated with reduced odds of resilience, with each unit increase in maternal or paternal MOPS score associated with a 3–4% decrease in the likelihood of ‘ACE-resilient’ trajectory membership (Mother: OR = 0.96, 95% CI: [0.93, 0.99]; Father: OR = 0.97, 95% CI: [0.95, 0.99]). Higher educational attainment was protective across both samples, conferring over a 35% increase in the odds of belonging to either higher well-being trajectory (‘ACE-resilient’: OR = 1.35, 95% CI: [1.15, 1.57]; ‘non-ACE-well’: OR = 1.36, 95% CI: [1.11, 1.68]).

Social connection was a robust predictor of well-being across samples. Socializing with friends one to two times more per week was associated with a ~50% increase in the odds of belonging to the higher well-being trajectory in both samples (‘ACE-resilient’: OR = 1.47, 95% CI: [1.18, 1.84]; ‘non-ACE-well’: OR = 1.53, 95% CI: [1.12, 2.08]). Social factors were particularly salient in the ACE sample, where being in a relationship more than doubled the likelihood of ‘ACE-resilient’ trajectory membership (OR = 2.23; 95% CI: [1.56, 3.18]). Although work absenteeism was not a significant predictor, higher self-rated work performance was associated with a ~ 30% increase in the odds of higher well-being trajectory membership in both samples (‘ACE-resilient’: OR = 1.35, 95% CI: [1.18, 1.53]; ‘non-ACE-well’: OR = 1.31, 95% CI: [1.10, 1.56]).

Within the health and lifestyle domain, poorer subjective physical health (SPHERE score) was associated with ~10% lower odds of higher well-being trajectory membership in both samples (‘ACE-resilient’: OR = 0.89, 95% CI: [0.86, 0.91]; ‘non-ACE-well’: OR = 0.90, 95% CI: [0.86, 0.94]). In the ACE sample only, higher BMI was associated with a ~ 25% reduction in the likelihood of ‘ACE-resilient’ membership (OR = 0.73; 95% CI: [0.59, 0.89]). Other lifestyle factors (sleep and physical activity) were not significant predictors. However, increased fruit and vegetable intake was associated with 41% higher odds of ‘non-ACE-well’ membership (OR = 1.41; 95% CI: [1.11, 1.80]). Alcohol consumption was also predictive in the non-ACE sample only, with moderate (weekly) drinkers, but not low or frequent drinkers, showing higher odds of ‘non-ACE-well’ membership relative to non-drinkers (OR = 3.00; 95% CI: [1.50, 5.99]). No effects were observed for smoking or substance use.

Across both samples, psychological factors showed consistent associations with well-being. A more internal locus of control, indexed by each unit increase in ICI score, was associated with a 6% increase in odds of higher well-being trajectory membership (‘ACE-resilient’: OR = 1.06, 95% CI: [1.03, 1.08]; ‘non-ACE-well’: OR = 1.06, 95% CI: [1.03, 1.09]). Higher neuroticism was associated with ~10% reduction in odds of higher well-being (‘ACE-resilient’: OR = 0.89, 95% CI: [0.87, 0.92]; ‘non-ACE-well’: OR = 0.91, 95% CI: [0.88, 0.95]), whereas higher extraversion was associated with an increase of over 10% in the odds of higher well-being across both samples (‘ACE-resilient’: OR = 1.15, 95% CI: [1.10, 1.20]; ‘non-ACE-well’: OR = 1.12, 95% CI: [1.08, 1.18]). In the ACE sample only, conscientiousness (OR = 1.07, 95% CI: [1.03, 1.11]) and openness (OR = 1.04, 95% CI: [1.01, 1.08]) were additionally associated with resilience. Emotional regulation strategies were not significantly associated with trajectory membership in either sample.

Results from the combined cross-domain models are presented in Supplementary Table 3 (ACE sample) and Supplementary Table 4 (non-ACE sample). In the ACE sample, educational attainment remained a unique predictor of ‘ACE-resilient’ membership (OR = 1.34, 95% CI: [1.08, 1.67]), alongside independent effects of psychological factors including lower neuroticism (OR = 0.90, 95% CI: [0.86, 0.94]), higher locus of control (OR = 1.06, 95% CI: [1.03, 1.08]), extraversion (OR = 1.18, 95% CI: [1.13, 1.24]), and conscientiousness (OR = 1.08, 95% CI: [1.03, 1.13]). Early environmental factors, social and occupational variables, health and lifestyle indicators, and openness did not independently predict resilience in the final model. Within the non-ACE sample, only positive psychological characteristics – locus of control (OR = 1.05, 95% CI: [1.01, 1.11]) and extraversion (OR = 1.17, 95% CI: [1.10, 1.32]) – remained independently associated with ‘non-ACE-well’ trajectory membership, with no independent effects observed for genetic, demographic, social, or health-related factors.

## Discussion

The present study examined whether a broad range of predictors across genetic, environmental, health, and psychological domains differentiated well-being trajectories across adulthood. Identifying pathways to resilience, a range of modifiable factors were associated with long-term well-being only among individuals exposed to ACEs. That is, beyond the social and work-related, health, and personality factors associated with well-being across samples, parenting, interpersonal relationships, and BMI were uniquely associated with resilience. This highlights the existence of shared and ACE-specific well-being resources that represent actionable targets for interventions designed to promote long-term well-being in both ACE-exposed individuals and the general population.

Of particular importance, adversity occurring within relationships – specifically interpersonal violation and peer conflict – emerged as potent predictors of reduced resilience. Maladaptive parenting, which was significantly more prevalent in individuals exposed to ACEs and is often itself considered an ACE (Anderson et al., 2022; Sasaki et al., 2024), further compounded this risk. Such interpersonal ACEs disrupt bonding and attachment during critical developmental periods and have been linked to long-term impairments in social functioning (Lüönd et al., 2022; Van Assche, Van de Ven, Vandenbulcke, & Luyten, 2020). These relational disruptions may underlie the elevated vulnerability to sustained lower well-being observed in the ACE sample, reinforcing the importance of early, relationship-focused interventions.

From a biological perspective, polygenic predisposition to well-being and family history of psychiatric illness were predictive of well-being in the non-ACE sample. This suggests that, in the absence of early adversity, individuals may be biologically primed toward higher or lower well-being, consistent with prior work linking genetic factors to life-course well-being (Baselmans et al., 2019; Jamshidi et al., 2020). In contrast, among individuals exposed to ACEs, neither genetic predisposition nor family history significantly predicted well-being, suggesting that early adversity may attenuate or override these biological influences. Importantly, even in the non-ACE sample where genetic effects were detectable, their magnitude was modest, indicating substantial scope for effective intervention. Moreover, the absence of effects for fixed characteristics such as age and biological sex in either sample further supports the view that well-being and resilience are not predetermined or static, but remain attainable across adulthood.

Educational attainment emerged as a particularly salient and leverageable well-being resource, associated with well-being across both samples. Further, it functioned as an independent predictor of resilience only among those individuals exposed to ACEs. ACEs such as household instability and peer conflict are known to undermine educational engagement and achievement (Qu et al., 2024; Schuurmans et al., 2022), which in turn constrain employment opportunities and long-term well-being (Armitage, Wootton, Davis, & Haworth, 2024; Baek et al., 2023). Supporting academic and vocational engagement may therefore represent a critical pathway through which well-being can be fostered, particularly in cases of early adversity.

Social relationships were also robust promoters of well-being. More frequent socializing with friends was associated with higher well-being across samples, supporting the social buffering hypothesis whereby emotional and practical support enhances mental health (Cheong, Sinnott, Dahly, & Kearney, 2017; Houghton et al., 2020). Further, more intimate interpersonal factors were uniquely salient in the ACE sample, with being in a relationship conferring additional resilience. Given evidence that ACEs disrupt attachment security and comfort with interpersonal closeness (Fitzgerald & Kawar, 2022; Lüönd et al., 2022), interventions that strengthen interpersonal skills and social resources may be particularly beneficial. Work performance similarly predicted well-being across samples, aligning with evidence that work contributes to meaning, motivation, and psychological health (Allan, Dexter, Kinsey, & Parker, 2018; Wang, 2023). Given the centrality of employment, enhancing work satisfaction may broadly confer well-being benefits across adulthood.

Health and lifestyle factors were also intertwined with mental well-being. Adverse physiological symptoms, such as muscle and joint pain, were consistently negatively associated with a high well-being trajectory. This highlights the importance of timely access to appropriate medical care as a pathway to both physical and psychological health and well-being. Lifestyle factors further contributed to well-being in the non-ACE sample, with higher fruit and vegetable intake predicting higher well-being. This adds to a growing body of evidence underscoring the importance of health-related lifestyle behaviors –particularly a balanced diet – for positive mental well-being (Muth, Losecaat Vermeer, Terenzi, & Park, 2022). This is especially relevant in the contemporary context of high food insecurity, itself a key contributor to both poor diet and lower well-being, which poses a barrier to accessing such nutrient-dense foods (Kent et al., 2024; Renard et al., 2024). These findings affirm the utility of community programs that improve access to fresh foods and promote their intake. The absence of this association in the ACE sample may reflect the established negative impact of early adversity on overall diet quality (Aquilina et al., 2021); dysregulated dietary consumption (such as nutritionally unbalanced meals and emotion-driven eating) may mask the potential well-being benefits of consistent fresh food intake.

Within the ACE sample alone, lower BMI was a significant predictor of higher well-being, with odds of ‘ACE-resilient’ membership decreasing by over 25% as BMI increased. This pattern aligns with evidence that prolonged early stress contributes to metabolic dysregulation and inflammation (Kurbatfinski, Dosani, Dewey, & Letourneau, 2024; Zagaria et al., 2024). Moreover, BMI has been shown to influence well-being even in the absence of overt physical illness, suggesting that psychosocial mechanisms such as weight-related discrimination may contribute to downstream mental health impacts (Casanova et al., 2021; Hunger & Major, 2015). ACEs themselves have been linked to heightened psychosocial consequences of weight, including exacerbated internalized weight stigma (Keirns et al., 2021), which may explain why the negative association between higher BMI and lower well-being was observed uniquely in the ACE sample. These findings underscore the importance of broader community-level interventions targeting factors such as discrimination alongside individual-level resources to foster sustained well-being.

Personality traits emerged as another consistent and independent predictor of long-term well-being. Extraversion and an internal locus of control were protective while neuroticism conferred risk, regardless of ACE exposure. These findings align with extensive evidence linking personality to well-being and stress reactivity (Musek, 2024; Wrzus, Luong, Wagner, & Riediger, 2021). Within the ACE sample, higher levels of conscientiousness and openness further distinguished resilient individuals. Prior work suggests exposure to ACEs may undermine levels of conscientiousness, with this trait mediating their negative effects on mental health (Koschig et al., 2023), whereas maintaining conscientiousness appears to support resilience (Lassen et al., 2022). Similarly, emerging evidence suggests openness may also contribute to resilience (Etilé, Frijters, Johnston, & Shields, 2021; Kang et al., 2023), possibly conferring psychological flexibility and adaptive coping. Together, these findings position personality as both a marker of risk and a viable intervention target, with growing evidence that traits can be modified through directed psychological interventions (Roberts et al., 2017). This could take the form of mindfulness-based interventions to facilitate the reduction of neuroticism and emotional reactivity (Almenräder, Heßmann, Voracek, & Tran, 2025) or strategies focused on building a growth mindset and to foster a more internal locus of control (Zappala-Piemme, Sturman, Brannigan, & Brannigan, 2023).

## Strengths and limitations

Key strengths of this study lie in its longitudinal, comprehensive assessment of well-being, enabling the identification of sustained, positively defined resilience across adulthood. Moreover, the inclusion of a broad range of predictors across biological, social, health, and psychological domains allowed for a nuanced examination of the multideterminant nature of well-being.

Several limitations should be acknowledged. First, while dichotomizing participants into ACE and non-ACE groups facilitated a targeted examination of resilience, it reduced sample sizes within each subgroup, potentially limiting power – particularly in the combined models with many predictors. In addition, because growth mixture models were estimated separately across groups, the resulting latent classes are not directly comparable. This precluded formal tests of group-by-predictor interactions and limited interpretations to descriptive comparisons. Second, ACEs were assessed using a binary endorsement approach, with any reported exposure defining ACE subsample membership. While single ACE exposures have been shown to be independently associated with adverse outcomes (e.g. Bellis, Hughes, Cresswell, & Ford, 2023; Kushner & Leban, 2024; Schilling, Aseltine, & Gore, 2007), this inclusive approach may have attenuated observed associations relative to higher cumulative thresholds. In addition, this measure did not capture the severity, frequency, or timing of adversity. Low prevalence of certain ACE types (e.g. exposure to warfare) also precluded examination of their specific impact. Future studies using more granular adversity measures in larger and more diverse samples are needed. Third, predictor variables were limited to those assessed at baseline, reflecting the prospective design. While this enables identification of early risk and protective factors associated with later well-being trajectories, it does not capture time-varying influences such as changes in relationships, environment, or behavior over time. Future studies incorporating repeated measures of these factors may provide more nuanced insight into dynamic resilience processes. Further, the restriction to individuals of European ancestry, necessary for genetic analyses, limited generalizability. Within the genetic analyses, associations between polygenic scores and well-being should be interpreted cautiously. Given the small and indirect effects typically observed for complex traits, and the potential that early adversity constrains environmental expression, the absence of association in the ACE-exposed group may reflect context-dependent attenuation of genetic influences rather than their absence. Exclusion of individuals with prior psychiatric or serious physical illness, while facilitating an examination of well-being in a nonclinical context, further limits generalizability. It may have also reduced variability in outcomes such as substance use and smoking, limiting the power to detect effects.

## Conclusions

This study demonstrates that although ACEs confer lasting risk for reduced well-being, resilience is both possible and underpinned by a constellation of protective factors. These include social connection, education, physical health, and modifiable personality characteristics, highlighting multiple accessible pathways to sustained well-being. Identifying these resources is central to empowering trauma-informed, strengths-based approaches that leverage such factors in the pursuit of lasting well-being in the face of early adversity.

## Supporting information

10.1017/S0033291726104784.sm001 Connon et al. supplementary material Connon et al. supplementary material

## Supplementary material

The supplementary material for this article can be found at http://doi.org/10.1017/S0033291726104784.

## Figure 1. Long description

This figure shows two panels of wellbeing trajectories across four time points (T1–T4), with wellbeing (COMPAS-W) scores on the y-axis (~80–110). The left panel presents the ACE-sample, where two trajectories are shown: a higher wellbeing “ACE-resilient” group (65.8%, n=585, solid line) and a lower wellbeing “ACE-risk” group (34.2%, n=304, dashed line). The right panel shows the non-ACE-sample, with a higher wellbeing “non-ACE-well” group (84.7%, n=660, solid line) and a lower wellbeing “non-ACE-vulnerable” group (15.3%, n=119, dashed line). In both panels, higher wellbeing groups remain consistently above lower wellbeing trajectories across time. Patterns are stable, with higher wellbeing trajectories maintaining scores around 100-105, and lower wellbeing trajectories maintaining scores around 88-90. Lines are smoothed estimates derived from longitudinal growth mixture modelling.

Navigate back to Figure 1..

## Table 1. Long description

This table shows within-domain logistic regression results for factors linked to resilient versus risk wellbeing trajectories in the ACE-sample (N=889). Significant childhood adversity predictors include interpersonal violation (OR=0.60), peer conflict (OR=0.64), and cumulative adversity (OR=0.88), all reducing resilience. Higher education (OR=1.35) increases resilience, while poorer maternal (OR=0.96) and paternal parenting (OR=0.97) reduce it. Social factors associated with resilience include being in a relationship (OR=2.23), socialising with friends (OR=1.47), and work performance (OR=1.35). Lower BMI (OR=0.73) and fewer physical health symptoms (OR=0.89) are also significant. Psychological traits linked to resilience include locus of control (OR=1.06), extraversion (OR=1.15), openness (OR=1.04), and conscientiousness (OR=1.07), while neuroticism (OR=0.89) reduces resilience. Other variables are not significant. Models adjusted for age, sex, zygosity, and family relatedness.

Navigate back to Table 1..

## Table 2. Long description

This table presents within-domain logistic regression results for factors associated with well versus vulnerable wellbeing trajectories in the non-ACE-sample (N=779). Higher polygenic score (OR=1.09) and educational attainment (OR=1.36) are linked to better wellbeing, while family history of mental illness (OR=0.53) reduces it. Socialising with friends (OR=1.53) and better work performance (OR=1.31) are positive predictors. Moderate alcohol intake (OR=3.00) and higher fruit and vegetable intake (OR=1.41) are also associated with better wellbeing, along with fewer physical health symptoms (OR=0.90). Psychological factors show higher locus of control (OR=1.06) and extraversion (OR=1.12) increase wellbeing, while neuroticism (OR=0.91) decreases it. Other variables are not significant. Models adjusted for age, sex, zygosity, and family relatedness.

Navigate back to Table 2..

## Figure 2. Long description

This figure is a forest plot displaying associations between childhood adversity subtypes and likelihood of belonging to the higher wellbeing (ACE-resilient) trajectory within the ACE-sample. The y-axis lists adversity types: personal health trauma, peer conflict, interpersonal violation, family illness/death/disaster, family breakup, and cumulative adversity score. The x-axis shows odds ratios on a logarithmic scale, with a dotted vertical line at OR=1 indicating no association. Points represent odds ratios and horizontal lines show 95% confidence intervals. Interpersonal violation, peer conflict, and cumulative adversity have odds ratios below 1 with confidence intervals not crossing 1, indicating significantly reduced likelihood of resilience. Other adversity types are not statistically significant. Models are adjusted for age, sex, zygosity, and family relatedness.

Navigate back to Figure 2..

## Figure 3. Long description

This figure presents two forest plots comparing predictors of higher wellbeing trajectory membership: ACE-resilient (left panel) and non-ACE-well (right panel). The y-axis lists predictors across five domains: genetics, demographic/environmental, social/employment, health/lifestyle, and psychological. The x-axis shows odds ratios on a logarithmic scale, with a dotted line at OR=1 indicating no association. Each predictor is represented by a point with 95% confidence intervals, and different shapes indicate domains (e.g., circle for genetics, square for demographic factors). Results mirror those in the tables: higher education, socialising with friends, work performance, and positive psychological traits (e.g., extraversion, locus of control) are associated with higher wellbeing, while neuroticism and adverse health indicators are linked to lower wellbeing. All models are adjusted for age, sex, zygosity, and family relatedness.

Navigate back to Figure 3..
