# Supplementary material for: Strengths-based resilience: the biopsychosocial factors that differentiate 12-year mental well-being trajectories following adverse childhood experiences
Source: Psychol Med. 2026 Jun 9;56:e185. doi: 10.1017/S0033291726104784 (PMC13247795; doi:10.1017/S0033291726104784)
Supplement: Connon et al. supplementary material [file S0033291726104784sup001.docx]

**Supplementary Material for**

**Strengths-based resilience: the biopsychosocial factors that differentiate 12-year mental wellbeing trajectories following adverse childhood experiences**

**Elizabeth Connon^1,2^; Haeme R.P. Park, PhD^2^, Robin M. Turner, PhD^3^, Leanne M. Williams, PhD^4^; Justine M. Gatt, PhD^1,2*^**

^1^School of Psychology, University of New South Wales, Sydney, NSW, Australia

^2^Neuroscience Research Australia, Randwick, NSW, Australia

^3^Biostatistics Centre, Division of Health Sciences, University of Otago, Dunedin, Central Dunedin, New Zealand

^4^Psychiatry and Behavioral Sciences, Stanford School of Medicine, Stanford University, Stanford, California, USA

**Correspondence:**

Justine Gatt, Adjunct Professor, School of Psychology, University of New South Wales

Email: [j.gatt@unsw.edu.au](mailto:j.gatt@unsw.edu.au)

**Supplementary Methods**

**Materials**

**Mental wellbeing.** Wellbeing was assessed at all timepoints with the COMPAS-W, a multidimensional measure of wellbeing comprising six subdomains (Composure, Own-worth, Mastery, Positivity, Achievement, Satisfaction) with high internal (0.84) and test-retest (0.82) reliability (Gatt et al., 2014). The COMPAS-W captures hedonic and eudaimonic aspects such as life satisfaction, positive affect, self-worth, agency, and life purpose.

**Adverse Childhood Experiences (ACEs).** Exposure to ACEs was measured retrospectively at baseline (T1) using the Early Life Stress Questionnaire (ELS-Q; Cohen et al., 2006), a tool developed from the Child Abuse and Trauma Scale (Sanders & Becker-Lausen, 1995). This questionnaire assesses for a broad range of adverse life experiences occurring in childhood that have been shown to potentially engender traumatic responses, and possesses high internal and criterion validity (Sokolowski et al., 2017)^.^ These events included physical, sexual, and emotional abuse, neglect, bullying, sustained family conflict, family separation, domestic violence, natural disaster, warfare, serious illness or injury, and death of family member. The item referring to ‘Premature birth’ was excluded from our analysis due to its high occurrence in twin births and non-psychological nature. Participants were categorised into either an ACE-exposed or a non-ACE-exposed sample for all analyses using their responses on this measure (ACE-sample, 'Yes' response to any items; non-ACE-sample, 'No' response to all items).

**Genetic predictors.** To assess potential genetic contributions to wellbeing and resilience, a polygenic score previously derived for the TWIN-10 sample was used. As described in Jamshidi et al. (2022), this score was constructed using genome-wide single nucleotide polymorphism (SNP) data and COMPAS-W scores as the target phenotype. The resulting polygenic score reflects the aggregate influence of many genetic variants associated with individual differences in this wellbeing phenotype.

**Demographic and environmental predictors.** A battery of self-report questionnaires was administered at baseline (T1), as described previously in the TWIN-10 protocol (Gatt et al., 2012). This captured demographic information including age, biological sex, family history of mental illness, and highest educational attainment (‘primary’, ‘secondary’, ‘post-secondary / trade’, ‘tertiary’, or ‘postgraduate’). Maladaptive parenting was assessed using the Measure of Parenting Style (MOPS), which demonstrates strong internal consistency (Cronbach’s α > 0.75 across subscales; Parker et al., 1997). The MOPS was completed separately for mothers and fathers and indexes negative caregiving experiences across dimensions of indifference, over-control and abuse.

**Social and employment predictors.** Relationship status was coded dichotomously as ‘in a relationship’ (‘married or defacto partnership’, ‘in a relationship’), or ‘not in a relationship’ (‘single’, ‘divorced’, ‘separated’). Social participation indexed the number of self-reported occasions per week participants typically socialised with others, assessed separately for interactions with family and friends. Employment-related outcomes were measured using the World Health Organisation Health and Work Performance Questionnaire (HPQ; Kessler et al., 2004). The HPQ has demonstrated reliability as a measure of work engagement (Cronbach’s α = 0.74; Golz et al., 2022) and was used to assess absenteeism (the discrepancy between expected and actual hours worked) and self-rated work performance (on a scale from 0-10).

**Health and lifestyle predictors.** A comprehensive medical history survey obtained height and weight (for calculation of body mass index; BMI) and average hours of sleep. Substance use was assessed via self-report, including alcohol consumption (‘never’, ‘low [monthly]’, ‘moderate [weekly]’, ‘frequent [>4 times per week]’), problematic substance use (response to whether a friend, relative or health professional had suggested reducing alcohol or drug use), and smoking status (‘never’, ‘former’, ‘current’). Physical activity was indexed as the number of weekly occasions participants engaged in strenuous exercise for at least 30 minutes. Dietary behaviour was assessed via self-reported weekly consumption of fruit and vegetables and fast food. Adverse subjective physical health was assessed using the Somatic and Psychological HEalth Report (SPHERE), which demonstrates high reliability in community samples (Cronbach’s α>0.85; Wijeratne et al., 2006). Sleep quality was assessed using 16 items derived from the Survey Screen for Apnea, designed to capture indicators of disrupted sleep (Cronbach’s α=0.85-0.93; Maislin et al., 1995).

**Psychological predictors.** Locus of control was measured using the Internal Control Index (ICI; Cronbach’s α=0.84; Duttweiler, 1984), with higher scores indicating a more internal locus of control and greater self-efficacy. Personality traits were assessed using the NEO Five-Factor Inventory (NEO-FFI), a 60-item measure of the five major personality domains: neuroticism, extraversion, openness, conscientiousness, and agreeableness (McCrae & Costa, 1987). Habitual emotion regulation strategies were assessed using the Emotion Regulation Questionnaire (ERQ), which measures cognitive reappraisal and expressive suppression (Cronbach’s α>0.73 for both subscales; Gross & John, 2003).

**Statistical Analysis**

**CATPCA of ACE items.** Within the ACE-sample only (N = 889), a categorical principal component analysis (CATPCA) on the ELS-Q items was conducted via SPSS (version 27; IBM Corp., Armonk, NY, USA). This was done to identify distinct co-occurring clusters of childhood adversity, facilitating an exploration of which particular forms of ACEs have the most notable impact on resilience. ELS-Q items reported by <2% of participants were removed ('Adoption', 'Home destroyed', 'Extreme Poverty', Warfare'), in line with recommended practice (Chu et al., 2013). To maximise the divergent validity and facilitate meaningful interpretation of each factor in the CATPCA, the Varimax rotation was applied. The Scree test and the criterion of an eigenvalue >1 were used to identify meaningful components, and items were included within the component onto which they loaded most strongly (Manisera, Kooij & Dusseldrop, 2010). As the purpose of this PCA was to identify co-occurring profiles of ACEs, components were retained regardless of contributing number of items. As displayed in Supplementary Table 1, the included 13 items yielded five components; 'Family breakup', 'Interpersonal violation’, 'Personal health trauma', 'Family illness, death and disaster', and 'Peer conflict’. Together, these components explained 55.53% of the total variance, and all items within a particular component had adequate factor loadings (>.40; Howard, 2016). The ACE subtypes derived from this process were used in subsequent regression analyses within the ACE-sample as predictors of wellbeing trajectory.

**Supplementary References**

Cohen, R. A., Hitsman, B. L., Paul, R. H., McCaffery, J., Stroud, L., Sweet, L., Gunstad, J., Niaura, R., MacFarlane, A., Bryant, R. A., & Gordon, E. (2006). Early life stress and adult emotional experience: An international perspective. *The International Journal of Psychiatry in Medicine, 36*(1), 35–52. doi:10.2190/5r62-9pqy-0nel-tlpa

Chu, D. A., Williams, L. M., Harris, A. W. F., Bryant, R. A., & Gatt, J. M. (2013). Early life trauma predicts self-reported levels of depressive and anxiety symptoms in nonclinical community adults: Relative contributions of early life stressor types and adult trauma exposure. *Journal of Psychiatric Research, 47*(1), 23–32. doi:10.1016/j.jpsychires.2012.08.006

Duttweiler, P. C. (1984). The Internal Control Index: A newly developed measure of locus of control. *Educational and Psychological Measurement, 44*(2), 209–221. doi:10.1177/0013164484442004

Gatt, J. M., Burton, K. L. O., Schofield, P. R., Bryant, R. A., & Williams, L. M. (2014). The heritability of mental health and wellbeing defined using COMPAS-W, a new composite measure of wellbeing. *Psychiatry Research, 219*(1), 204–213. doi:10.1016/j.psychres.2014.04.033

Gatt, J. M., Korgaonkar, M. S., Schofield, P. R., Harris, A., Clark, C. R., Oakley, K. L., Ram, K., Michaelson, H., Yap, S., Stanners, M., Wise, V., & Williams, L. M. (2012). The TWIN-E project in emotional wellbeing: Study protocol and preliminary heritability results across four MRI and DTI measures. *Twin Research and Human Genetics, 15*(3), 419–441. doi:10.1017/thg.2012.12

Golz, C., Gerlach, M., Kilcher, G., & Peter, K. A. (2022). Cultural adaptation and validation of the Health and Work Performance Questionnaire in German. *Journal of Occupational and Environmental Medicine, 64*(12), e845–e850. doi:10.1097/jom.0000000000002719

Gross, J. J., & John, O. P. (2003). Individual differences in two emotion regulation processes: Implications for affect, relationships, and well-being. *Journal of Personality and Social Psychology, 85*(2), 348–362. doi:10.1037/0022-3514.85.2.348

Howard, M. C. (2016). A review of exploratory factor analysis decisions and overview of current practices: What we are doing and how can we improve? *International Journal of Human–Computer Interaction, 32*(1), 51–62. doi:10.1080/10447318.2015.1087664

Jamshidi, J., Schofield, P. R., Gatt, J. M., & Fullerton, J. M. (2022). Phenotypic and genetic analysis of a wellbeing factor score in the UK Biobank and the impact of childhood maltreatment and psychiatric illness. *Translational Psychiatry, 12*(1), 113. doi:10.1038/s41398-022-01874-5

Kessler, R. C., Ames, M., Hymel, P. A., Loeppke, R., McKenas, D. K., Richling, D. E., Stang, P. E., & Ustun, T. B. (2004). Using the World Health Organization Health and Work Performance Questionnaire (HPQ) to evaluate the indirect workplace costs of illness. *Journal of Occupational and Environmental Medicine, 46*(6 Suppl), S23–S37. doi:10.1097/01.jom.0000126683.75201.c5

Maislin, G., Pack, A. I., Kribbs, N. B., Smith, P. L., Schwartz, A. R., Kline, L. R., Schwab, R. J., & Dinges, D. F. (1995). A survey screen for prediction of apnea. *Sleep, 18*(3), 158–166. doi:10.1093/sleep/18.3.158

Manisera, M., van der Kooij, A. J., & Dusseldorp, E. (2010). Identifying the component structure of satisfaction scales by nonlinear principal components analysis. *Quality Technology & Quantitative Management, 7*(1), 97–115. doi:10.1080/16843703.2010.11673222

McCrae, R. R., & Costa, P. T. (1987). Validation of the five-factor model of personality across instruments and observers. *Journal of Personality and Social Psychology, 52*(1), 81–90. doi:10.1037/0022-3514.52.1.81

Sanders, B., & Becker-Lausen, E. (1995). The measurement of psychological maltreatment: Early data on the child abuse and trauma scale. *Child Abuse & Neglect, 19*(3), 315–323. doi:10.1016/S0145-2134(94)00131-6

Sokolowski, A., & Dragan, W. L. (2017). New empirical evidence on the validity and the reliability of the early life stress questionnaire in a Polish sample. *Frontiers in Psychology, 8*, 365. doi:10.3389/fpsyg.2017.00365

Parker, G., Roussos, J., Hadzi-Pavlovic, D., Mitchell, P., Wilhelm, K., & Austin, M. P. (1997). The development of a refined measure of dysfunctional parenting and assessment of its relevance in patients with affective disorders. *Psychological Medicine, 27*(5), 1193–1203. doi:10.1017/S003329179700545X

Wijeratne, C., Hickie, I., & Davenport, T. (2006). Is there an independent somatic symptom dimension in older people? *Journal of Psychosomatic Research, 61*(2), 197–204. doi:10.1016/j.jpsychores.2006.01.012

**Supplementary Tables**

**Supplementary Table 1.** Factor loadings for the five components derived from the CATPCA of the ELS-Q in the ACE-sample (N = 889).

| **ELS-Q item** | **Family breakup** | **Interpersonal violation (IPV)** | **Personal health trauma** | **Family illness, death and disaster** | **Peer conflict** |
| --- | --- | --- | --- | --- | --- |
| **Sustained family conflict** | .785 |  |  |  |  |
| **Family separation** | .692 |  |  |  |  |
| **Physical abuse** |  | .755 |  |  |  |
| **Emotional abuse** |  | .684 |  |  |  |
| **Sexual abuse** |  | .609 |  |  |  |
| **Domestic violence in the family** |  | .486 |  |  |  |
| **Major surgery / hospitalisation** |  |  | .810 |  |  |
| **Illness / injury** |  |  | .807 |  |  |
| **Family death** |  |  |  | .691 |  |
| **Family illness** |  |  |  | .684 |  |
| **Natural disaster** |  |  |  | .418 |  |
| **Sustained bullying or rejection by schoolmates** |  |  |  |  | .945 |
| **Eigenvalue** | 1.82 | 1.80 | 1.33 | 1.24 | 1.03 |
| **% of variance in ELS-Q explained (Total = 55.53%)** | 14.03 | 13.86 | 10.25 | 9.50 | 7.90 |

*Note.* Factor loadings from categorical principal component analysis (CATPCA), with the Varimax rotation applied. ELS-Q, Early Life Stress – Questionnaire.

**Supplementary Table 2.** Baseline Demographic Characteristics for Individuals with and without Adverse Childhood Experiences (ACEs)

|  | **With ACEs**  **n = 889** | **Without ACEs**  **n = 779** | **Test statistic** | ***p* value** |
| --- | --- | --- | --- | --- |
|  | **N (%)** | **N (%)** |  |  |
| **Categorical variables** |  |  |  |  |
| **Sex** |  |  | *X*^2^ = 3.55 | 0.06 |
| Female | 504 (57%) | 478 (61%) |  |  |
| Male | 385 (43%) | 301 (39%) |  |  |
| **Relationship status** |  |  | *X*^2^ = 3.72 | 0.16 |
| In a relationship | 543 (61%) | 502 (64%) |  |  |
| Divorced / separated | 70 (8%) | 44 (6%) |  |  |
| Single | 263 (30%) | 229 (29%) |  |  |
| Widowed | 4 (1%) | 3 (1%) |  |  |
| **Highest education** |  |  | *X*^2^ = 7.69 | 0.05 |
| Secondary school | 232 (26%) | 211 (27%) |  |  |
| Post-school diploma / qualification | 268 (30%) | 194 (25%) |  |  |
| Bachelor's degree | 222 (25%) | 231 (30%) |  |  |
| Postgraduate degree | 160 (18%) | 136 (17%) |  |  |
| Other | 7 (<1%) | 7 (<1%) |  |  |
| **Family history of mental illness** |  |  | *X*^2^ = 0.14 | 0.71 |
| Yes | 82 (9%) | 69 (9%) |  |  |
| No | 480 (54%) | 438 (56%) |  |  |
| Unsure or prefer not to say | 327 (37%) | 272 (45%) |  |  |
| **Continuous variables** | **Mean (SD)** | **Mean (SD)** |  |  |
| Age | 39.70 (12.70) | 39.60 (12.8) | *t* = -0.22 | 0.83 |
| Wellbeing (COMPAS-W) | 98.40 (10.90) | 102.00 (9.74) | ***t* = 6.46** | **<0.001** |
| MOPS – mother | 4.54 (5.63) | 2.39 (3.12) | ***t* = -9.83** | **<0.001** |
| MOPS – father | 5.53 (7.25) | 2.44 (3.70) | ***t* = -11.2** | **<0.001** |

*Note*: Data presented from baseline measurement. Bolding indicates a statistically significant difference between samples.

Abbreviations: COMPAS-W, Composure, Own-worth, Mastery, Positivity, Achievement, Satisfaction – Wellbeing scale; MOPS, Measure of Parenting Style.

**Supplementary Table 3**. Combined, cross-domain associations between significant variables and wellbeing trajectories (Resilient vs Risk) in the ACE sample (*N* = 889).

|  | **Resilient (vs Risk)** | | |
| --- | --- | --- | --- |
| **Variable** | **Odds ratio** | **95% CIs** | ***P* value** |
| ACEs – Interpersonal Violation | 0.67 | 0.37, 1.22 | 0.19 |
| ACEs – Peer conflict | 0.83 | 0.52, 1.33 | 0.44 |
| ACEs – cumulative count | 1.04 | 0.86, 1.26 | 0.69 |
| MOPS – Mother | 1.03 | 0.99, 1.08 | 0.15 |
| MOPS – Father | 0.98 | 0.94, 1.01 | 0.18 |
| Educational attainment | **1.34** | **1.08, 1.67** | **0.008** |
| Relationship status (*reference = in a relationship)* | 1.37 | 0.85, 2.23 | 0.20 |
| Socialising with friends | 1.05 | 0.78, 1.15 | 0.75 |
| Work performance (HPQ) | 0.95 | 0.79, 1.15 | 0.58 |
| BMI (categories) | 0.89 | 0.67, 1.18 | 0.42 |
| Adverse physical health symptoms (SPHERE) | 0.99 | 0.95, 1.03 | 0.65 |
| Locus of Control (ICI) | **1.06** | **1.03, 1.08** | **<.001** |
| Neuroticism (NEO-FFI) | **0.90** | **0.86, 0.94** | **<.001** |
| Extraversion (NEO-FFI) | **1.18** | **1.13, 1.24** | **<.001** |
| Openness (NEO-FFI) | 1.03 | 0.99, 1.07 | 0.17 |
| Conscientiousness (NEO-FFI) | **1.08** | **1.03, 1.13** | **0.001** |

*Note:* Analysis adjusted for age, sex, zygosity and family relatedness. Bolding indicates a statistically significant difference between the wellbeing trajectories.

Abbreviations: MOPS, Measure of Parenting Style; HPQ, Health and Work Performance Questionnaire; BMI, Body Mass Index; SPHERE, Somatic and Psychological HEalth REport; ICI, Internal Control Index; NEO-FFI, NEO Five Factor Inventory.

**Supplementary Table 4**. Combined, cross-domain associations between significant variables and wellbeing trajectories (Well vs Vulnerable) in the non-ACE sample (*N* = 779).

|  | **Well (vs Vulnerable)** | | |
| --- | --- | --- | --- |
| **Variable** | **Odds ratio** | **95% CIs** | ***P* value** |
| Polygenic score | 1.04 | 0.97, 1.12 | 0.27 |
| Family history of mental illness | 0.56 | 0.20, 1.57 | 0.27 |
| Educational attainment | 1.21 | 0.83, 1.76 | 0.33 |
| Socialising with friends | 1.19 | 0.75, 1.90 | 0.46 |
| Work performance (HPQ) | 1.03 | 0.78, 1.36 | 0.85 |
| Adverse physical health symptoms (SPHERE) | 0.94 | 0.88, 1.01 | 0.11 |
| Alcohol intake *(reference = never)* | | | |
| Low (monthly) | 2.40 | 0.63, 6.45 | 0.076 |
| Moderate (less than weekly) | 2.99 | 0.87, 1.18 | 0.06 |
| Frequent (weekly) | 2.18 | 0.89, 3.03 | 0.21 |
| Fruit and vegetable intake | 1.32 | 0.87, 1.99 | 0.19 |
| Locus of Control (ICI) | **1.05** | **1.01, 1.11** | **0.006** |
| Neuroticism (NEO-FFI) | 0.94 | 0.86, 1.00 | 0.05 |
| Extraversion (NEO-FFI) | **1.17** | **1.10, 1.32** | **<.001** |

*Note:* Analysis adjusted for age, sex, zygosity and family relatedness. Bolding indicates a statistically significant difference between the wellbeing trajectories.

Abbreviations: HPQ, Health and Work Performance Questionnaire; SPHERE, Somatic and Psychological HEalth REport; ICI, Internal Control Index; NEO-FFI, NEO Five Factor Inventory.
